# Multiple Sclerosis Patients with Markedly Low Intrathecal Antibody Response in Sri Lanka

**DOI:** 10.1155/2018/5342936

**Published:** 2018-02-28

**Authors:** S. M. K. Gamage, I. Wijeweera, S. B. Adikari, Katharina Fink, Jan Hillert, Anna Fogdell-Hahn, H. M. A. Sominanda

**Affiliations:** ^1^Department of Anatomy, Faculty of Medicine, University of Peradeniya, Peradeniya, Sri Lanka; ^2^Neurology Unit, Teaching Hospital, Kandy, Sri Lanka; ^3^Department of Neurology, Karolinska University Hospital, Stockholm, Sweden; ^4^Department of Clinical Neuroscience, Karolinska Institutet, Stockholm, Sweden

## Abstract

Multiple sclerosis (MS) is a heterogeneous disease which is poorly studied in Asia, where the disease is known to be rare with significant differences in clinical and radiological presentations and intrathecal antibody response. Therefore the objective of this study was to determine clinical presentation, radiological and neurophysiological characteristics, and oligoclonal band status in Sri Lankan MS patients, following careful exclusion of patients with neuromyelitis optica spectrum disorders and other conditions mimicking multiple sclerosis. Sixty-nine MS patients were recruited to the study adhering to McDonald 2010 criteria. Their clinical presentation, characteristics of central nervous system lesions in magnetic resonance imaging, visual evoked potential (VEP) results, oligoclonal bands (OCB), and AQP4 antibody status were studied. Of 69 MS patients, 54%, 6%, and 1% were relapsing remitting, secondary progressive, and primary progressive, respectively, and 39% were patients with clinically isolated syndrome. The commonest clinical presentations were cerebral motor followed by cerebral sensory and optic neuritis. Majority had typical periventricular and infratentorial lesions in MRI. Though not clinically apparent, bilateral delay of P100 wave latency was present in 52%. OCB positivity was 42% and AQP4 antibody was positive in only one patient. In conclusion, this group of Sri Lankan MS patients shares most of the clinical and radiological features of Caucasian MS patients. However, the OCB positivity is lower in this group, when compared to the Caucasian MS populations.

## 1. Background

Multiple sclerosis (MS) is heterogeneous in terms of disease prevalence, clinical and paraclinical characteristics in different regions, and ethnicities in the world [1–4]. Even though the precise reasons for this heterogeneity have not been identified yet, geographical and genetic influences have long been hypothesized as causes [5]. There is higher prevalence of MS in countries away from equator than in regions close to equator. In addition there is very high prevalence of MS in ethnic groups such as Sardinians and Parsis, while there is low prevalence among Samis, Turkman, Uzbeks, native Siberians, and New Zealand Maoris [6]. There are MS hot spots in Florida, Canada, and Norway, reinforcing the fact that geographic factors may have directly influenced occurrence of MS [6, 7]. This is probably due to the geographic isolation of populations causing selection of candidate genes responsible for MS. This aspect may be relevant to Sri Lanka which is a geographically isolated island in which the unique geography can influence its confined population genetics. However, there are anthropological evidence which suggests that Sri Lanka has a mixed genetic composition due to the significant genetic flow from its neighboring country India [8]. Nevertheless, it was recently proved that Sri Lankans are different to Caucasians in clinical pathologies, disease courses, outcomes, and treatment responses for many diseases [9].

Multiple sclerosis is poorly studied in Sri Lanka. The exact prevalence of MS is not studied up to now. However, there is handful of studies on MS in the South Asian region, including Sri Lanka [10, 11]. Most of them have reported markedly low disease prevalence and inconsistent accounts on the clinical picture of MS. Most importantly, the paraclinical characteristics such as magnetic resonance imaging (MRI) features, oligoclonal band (OCB) status, and neurophysiological investigation results have not been studied adequately in this region of the world. This study is the first comprehensive descriptive study on the largest sample of MS patients in Sri Lanka, especially with the OCB status.

## 2. Methods

### 2.1. Aim

Based on the hypothesis that Sri Lankan MS patients are different to Caucasian patients, the objective of this study was to characterize MS in Sri Lanka using clinical and paraclinical findings, following meticulous exclusion of all cases of neuromyelitis optica spectrum disorder (NMOSD) and other conditions mimicking MS.

### 2.2. Patients, Study Design, and Setting

This is a cross-sectional descriptive study. Ethical approval was granted by the Faculty of Medicine, Peradeniya (2012/EC/14). Patients referred from 5 main referral hospitals, representing 8 out of 9 provinces of Sri Lanka, during the period of March 2012 to December 2015 were included in the study with informed written consent. Patients were diagnosed by consultant neurologists using clinical, MRI, and visual evoked potential (VEP) parameters. The following were the inclusion and exclusion criteria for patient recruitment of the study.

*Inclusion Criteria*. The inclusion criteria are as follows: patients fulfilling McDonald 2010 criteria (both newly diagnosed patients and patients presenting with relapses); availability of at least one MRI with T1W/T2W/FLAIR pulse sequence images; availability of at least one MRI reported by consultant radiologist; patients with informed written consent for lumbar puncture and availability of appropriate samples of cerebrospinal fluid (CSF) and serum for OCB and aquaporin 4 antibody (AQP4) assays.

*Exclusion Criteria*. The exclusion criteria are as follows: NMOSDs diagnosed according to revised Wingerchuk criteria 2015 following AQP4 antibody analysis; patients having recurrent myelitis with longitudinally extensive spinal cord lesions that contiguously extend over 3 vertebral segments; patients with acute disseminated encephalomyelitis (ADEM), vasculitis, cerebrovascular accidents, and other conditions mimicking MS; patients with disease duration less than 12 months; patients with disease onset earlier than 12 years and later than 60 years; patients with diagnosed and uncontrolled psychiatric disorders and prominent grey matter disorders such as dementia and seizures.

### 2.3. Data Retrieval and Collection

The clinical and investigation results were initially collected locally at the referring hospitals in a retrospective manner from the bed head records and clinical records of all patients. Whenever there was a lack of availability of data in patients' records, the principle investigator interviewed and examined patients individually to obtain more detailed information. These details were documented in a structured data collection sheet until further analysis.

All the MRIs obtained at the onset of disease and up to a maximum of 5 relapses were considered, if they fulfilled inclusion criteria. Details in MRI reports issued by Consultant Radiologists and data in the most recent CSF and VEP reports were extracted and stored in the structured data collection sheet.

### 2.4. Biological Samples

Paired serum and CSF samples from each patient were obtained for OCB and AQP4 antibody assays at referring hospitals, during diagnostic assessment in CIS patients and during a relapse in already diagnosed MS patients. All samples were transported to the Faculty of Medicine, University of Peradeniya, at a temperature of 2–8°C, within 24 hours of collection. These samples were aliquoted and stored at −45°C and the final analysis was performed within three months of sample acquisition in the Faculty of Medicine, University of Peradeniya.

### 2.5. MRI Brain and Spinal Cord

All MRI reports of brain and spinal cord, obtained during the onset of disease and up to a maximum of 5 relapses, were considered, only if they had been reported by the Consultant Radiologists. Data regarding the location, size, lesion number, and T1, T2, FLAIR intensities, and contrast enhancements and presence and absence of new lesions were extracted and recorded in the data collection sheet until further analysis.

### 2.6. Laboratory Analyses: Isoelectric Focusing and Immunoblotting (IEF and IB) for OCB Detection in CSF and Serum

A validated IEF and immunoblotting protocol for detection of OCB, adopted in Karolinska Institutet Sweden [13], was established in the immunology laboratory, Faculty of Medicine, University of Peradeniya. Twenty OCB positive and negative CSF and serum control samples of western MS patients were obtained from Karolinska Institutet. These samples were used as positive and negative controls during OCB analysis in our laboratory.

### 2.7. ELISA for AQP4 Antibody Detection

Serum AQP4 antibody was tested in all patients using the Aquaporin-4 Ab version 2 autoantibody kit (RSR limited, Cardiff, UK), according to manufacturer's protocol.

### 2.8. Data and Statistical Analysis

Data retrieved from the data collection sheets were entered into Microsoft Excel and were analyzed using Graph Pad Prism software (Graph Pad Software, La Jolla California USA, https://www.graphpad.com).

Since no data from the control Caucasian MS group were available, no formal statistical comparison could be conducted between Sri Lankan and Caucasian MS patients.

Descriptive statistics was applied for the Sri Lankan MS group and the comparison with Caucasian MS was based on the data from literature.

## 3. Results

### 3.1. Study Population and Definitions

**Figure 1:**
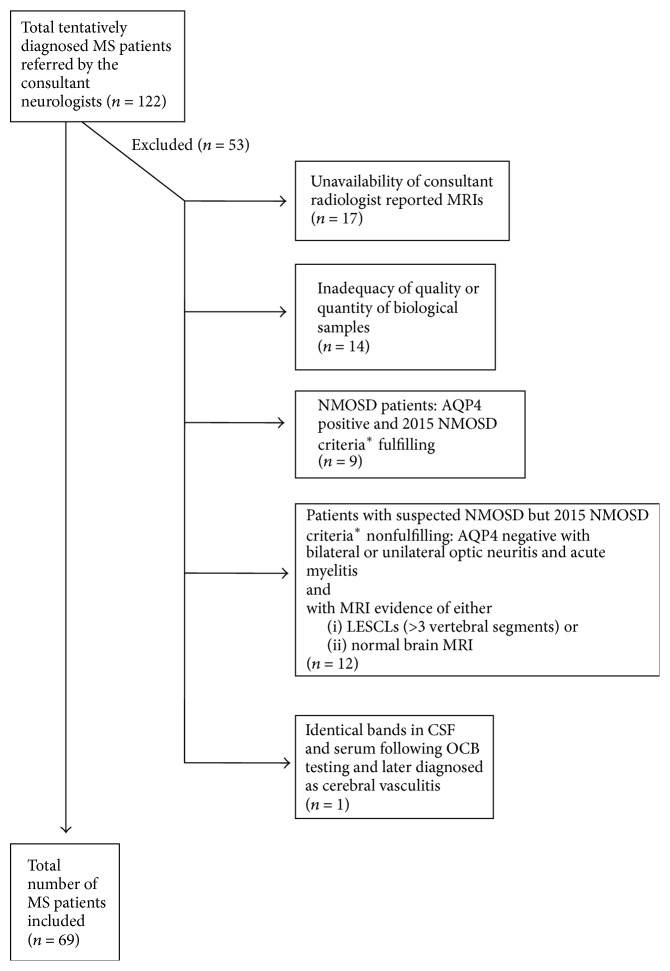
*Sample selection*. ^*∗*^2015 NMOSD criteria defined by Wingerchuk et al. [12].

Following application of inclusion and exclusion criteria, 69 MS patients were included in the study (Figure 1). Of them, 54%, 6%, 1%, and 39% were categorized as relapsing remitting (RRMS), secondary progressive (SPMS), primary progressive (PPMS), and clinically isolated syndrome (CIS), respectively. The demographic characteristics are shown in Table 1.

### 3.2. Clinical Characteristics

The commonest clinical presentation was cerebral motor manifestations (50%) followed by cerebral sensory (45%) and optic neuritis (45%). Cerebellar and brain stem manifestations were present in only 32% and 29%, respectively. Spinal cord sensory and motor manifestations were observed in 19% of patients. Autonomic manifestations were observed in 8% while fatigue was observed in 48% (Table 2).

**Figure 2:**
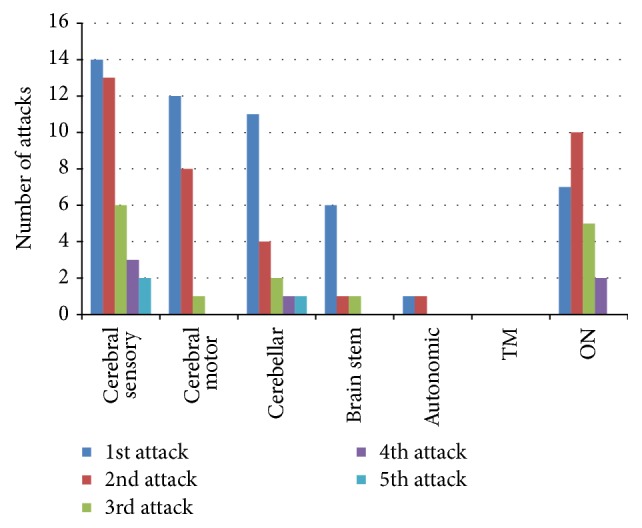
*Frequency of different clinical manifestations in the study group*. TM: transverse myelitis; ON: optic neuritis.

Out of the total number of relapses, the cerebral sensory relapses were the highest compared to the cerebral motor, cerebellar, optic neuritis, or autonomic relapses. Out of all clinical manifestations, cerebral sensory manifestations predominated during the 1st, 2nd, and 3rd relapses followed by optic neuritis. Thus, even though cerebral motor manifestations were the most common initial clinical presentation, it was the 3rd frequent clinical feature during relapses (Figure 2).

### 3.3. Psychosocial Manifestations

Depression secondary to MS was diagnosed in 16%. Suicidal thoughts were present in 16% of the patients with MS though there were no suicidal attempts reported (Table 3).

### 3.4. MRI Features

**Figure 3:**
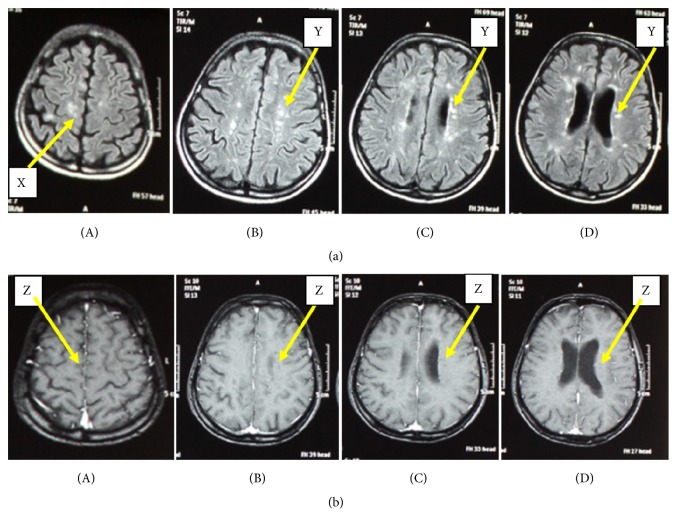
*FLAIR hyperintense and T1W hypointense lesions in cerebrum of an MS patient*. (a) MRIs of McDonald criteria fulfilling MS patient showing sections of cerebrum with hyperintense lesions in FLAIR sequence; (a)(A) oval and round shaped subcortical lesions indicated by “X”; (a)(B), (a)(C), and (a)(D) oval periventricular lesions located perpendicular to the ventricular surface indicated by “Y” (Dawson's fingers appearance). (b) MRIs of a patient showing sections of cerebrum with hypointense lesions in T1W sequence; (b)(A) hypointense lesions in subcortical region indicated by “Z”; (b)(B), (b)(C), and (b)(D) hypointense lesions in periventricular regions indicated by “Z”.

**Figure 4:**
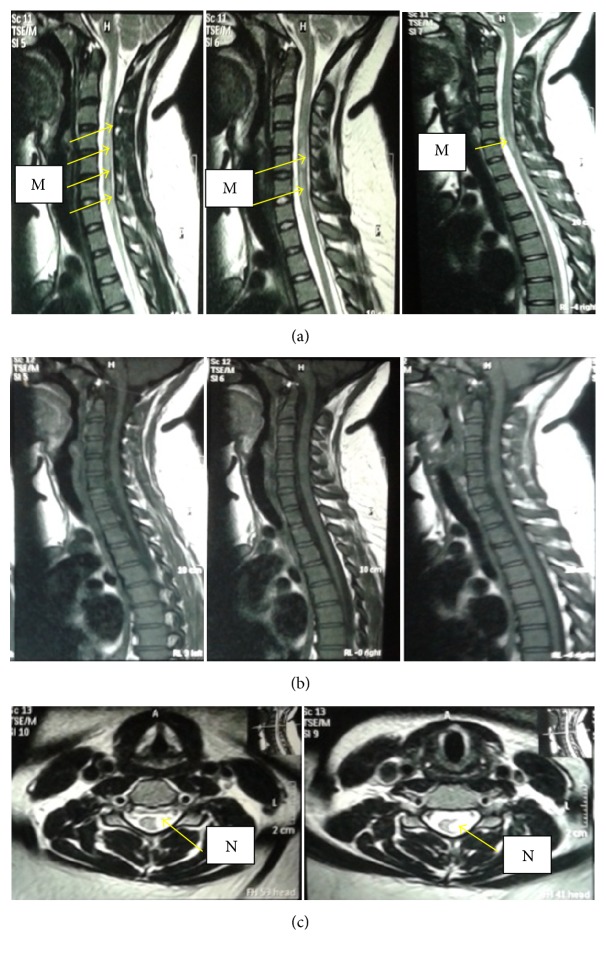
*T2W hyperintense and T1W hypointense lesions in spinal cord of an MS patient*. (a) MRIs showing three sagittal sections of spinal cord with patchy, hyperintense lesions in T2W sequence indicated in M. (b) MRIs showing three sagittal sections of spinal cord with hypointensities in T2W sequence. (c) MRIs of the same patient showing two transverse sections of spinal cord with peripherally located hyperintense lesion in T2W sequence.

All patients demonstrated CNS lesions, which are hyperintense in T2W and FLAIR images and hypointense in T1W images. The periventricular lesions were perpendicular to the ventricular surfaces (Figure 3) and were present in 80% of patients (Table 4). Juxtacortical lesions were seen in 69%. Infratentorial and corpus callosal lesions were present in 74% and 49%, respectively. Spinal cord and cervical cord lesions were patchy and peripherally located (Figure 4).

### 3.5. Clinical Optic Neuritis (ON) and VEP Assessment

Clinically monocular optic neuritis was commoner than binocular optic neuritis in this group of MS patients. VEP reports were available in 80 eyes of 40 MS patients. Of those, majority showed bilateral P100 wave latency delay (Table 5). Most importantly, out of the 18 clinically ON negative eyes, 6 had P100 wave latency delay reflecting existence of subclinical optic nerve demyelination.

### 3.6. CSF Findings

CSF full reports were available in 80% of the patients. Mean cell count was 26 (±12). Oligoclonal bands were positive in 42% of the group (Table 6).

### 3.7. AQP4 Antibody Status

Of all MS patients (*n* = 69), 99% were negative for AQP4 antibodies and only one patient was positive for AQP4 antibodies (Table 6).

## 4. Discussion

This is the first study done on a large group of MS patients of a country situated in a region with low prevalence of MS [14]. This group of Sri Lankan MS patients shows considerable overlapping with Caucasian MS patients clinically and radiologically, but there is a striking difference with respect to OCB status.

### 4.1. Demographics and Clinical Characteristics

This group showed a female preponderance with a male to female ratio of 1 : 1.7 similar to studies done in Europe, Asia, and elsewhere in the world [15–19]. In addition, mean age at disease onset was also comparable with the studies in Asia as well as in Caucasian populations [20]. Clinical subtype of the majority of this group was RRMS and there were a considerable number of CIS cases. However, the percentages of SPMS and PPMS cases were less when compared to similar studies [21, 22]. A probable reason could be inclusion of patients at an earlier stage of the disease.

The relapse frequency was higher than the Caucasian and Japanese MS patients reflecting a higher disease activity [23]. This may be explained by the shorter duration of disease of this study group, since disease activity is known to be high during the initial stage of the disease [24]. However, the mean EDSS of this cohort is 2.5, which is a relatively mild disability compared to most of the Japanese studies [25] indicating a lower neurological deficit due to a lesser disease progression. Furthermore, MSSS (multiple sclerosis severity score) and Global ARMSSS (age related multiple sclerosis severity score) were 5.7 and 5.3, respectively, in the present study group [26]. These values are comparable, yet lower than those of the Global MSSS and Global ARMSSS matrix data (6.37 and 5.93, resp.), which have been developed using data on European MS patients [26].

The commonest initial clinical presentation was cerebral motor manifestations followed by cerebral sensory features and optic neuritis (Table 2). A similar pattern has been observed in a previous study done on Sri Lankan MS and in other Asian studies [10, 27]. On the contrary, studies on Caucasian MS patients have demonstrated that sensory presentations predominate over motor manifestations [4, 28]. However, as illustrated in Figure 2, majority of clinical attacks of these MS patients consisted of sensory manifestations, followed by optic neuritis and motor manifestations which is on par with Caucasian MS [28]. Patients are more likely to consult a neurologist following the first motor symptoms rather than sensory symptoms, since sensory manifestations are not as disabling as motor. Therefore the reasons for most initial presentations to be motor but majority of relapses to be sensory may be that the patients might have ignored the initial sensory symptoms or may be the initial sensory symptoms had not been recognized as a result of underlying demyelinating disease.

Bilateral severe optic neuritis is commonly considered as a main clinical presentation of NMO as opposed to the unilateral optic neuritis which is a common presentation in early MS [12] and this study reports a similar pattern. Furthermore, none of the patients had complete loss of light perception, indicating a less severe form of demyelination in the optic pathway. Internuclear ophthalmoplegia (INO) is a common occurrence in MS and it is reported to be present in up to 30% of MS patients [29]. The present study has demonstrated a similar frequency. Lhermitte's sign, a pathognomonic clinical feature in western MS [28], was as low as 19% in Sri Lankan* “MS”* patients. In addition, a lower occurrence of cerebellar, brainstem/cranial nerve palsies and autonomic manifestations were observed when compared with the studies on Caucasian MS patients [30].

Depression and fatigability appear to be common in Sri Lankan MS patients as reported elsewhere in the world [31, 32]. Suicidal thoughts, loss of libido, divorce related to the disease, and loss of employment were also found to be substantially high among MS patients indicating a negative impact on the psychosocial aspects of these patients. There was no positive family history of MS reported in any of the patients in this study reflecting the sporadic nature of the disease in Asia, in contrast to the familial nature of MS in temperate countries [33, 34].

### 4.2. Imaging and Neurophysiological Characteristics

All of the MS patients had round or oval shaped lesions (Figures 3 and 4) scattered in neuroanatomical locations typical for MS [30] (Table 4). Of these, periventricular lesions were perpendicular to ventricular surface indicating “Dawson finger appearance” which is a typical finding in Caucasian MS patients [35] (Figure 3). Occurrence of infratentorial lesions in MRI is reported to be very common in Caucasian MS patients when compared to Asian MS patients [36]. However in the present study, the majority had infratentorial lesions typical for MS indicating their close resemblance to Caucasian MS patients. Similarly, patchy and peripherally located spinal cord lesions, which do not contiguously extend more than 3 vertebral segments, are considered to be typical of conventional Caucasian type MS and it is an important MRI finding for the differentiation of MS from NMOSDs [12]. Such spinal cord lesions (including cervical cord lesions) were found in a considerable number of patients in the present study group. Therefore this group of Sri Lankan MS patients resembles the Caucasian type of MS radiologically.

Monocular optic neuritis (ON) is very common among Sri Lankan MS patients, confirmed by P100 wave latency delay. However, there were a large number of eyes without clinical ON but with P100 wave latency delay indicating subclinical evidence of optic nerve demyelination. Similar findings have been reported in literature on Caucasian MS, substantiating the clinical and MRI resemblance of Sri Lankan MS group to those of Caucasian MS [37].

### 4.3. Intrathecal Cellular and Antibody Response

Lower OCB positivity (42%) was observed in this group of Sri Lankan MS patients when compared to that of Caucasian MS patients (95–98%) [38]. However this value is similar to the available Japanese and Korean studies performed using the same standard method [17, 25]. None of the Asian studies have reported OCB positivities as high as 90% up to now. Therefore the reason for low OCB positivity in this region of the world can be a difference in intrathecal antibody response of the Asian MS patients. The clinical features of Asian and Caucasian MS patients seem to overlap, but due to the differences in intrathecal antibody responses the OCB positivity may vary in different populations. This may be attributed to differences in the B cell or humorally mediated immunopathogenesis of MS in the Asian patients. Further research on this possibility is necessary to speculate the cause for the lower OCB positivity among Sri Lankan as well as other Asian MS patients. Alternatively, the CIS patients in the present study who are at the initial stage of their disease may have a possibility of seroconversion to OCB positivity in later course of the disease [39].

A Swedish study done on 1505 MS patients concluded that OCB negative subpopulation was clinically indistinguishable from the OCB positive group, since they shared most of the clinical features. OCB positive MS patients' genetic makeup has been found to be consisting of more HLA-DRB1*∗*15 genotype while that of OCB negative patients has been associated more with HLA-DRB1*∗*04 genotype [13]. Therefore, as a future direction, it is important to explore the HLA associations of the OCB positive and negative subgroups in Sri Lanka.

There was one patient repeatedly positive for AQP4 in this group and he had unilateral optic neuritis, cerebral sensory manifestations, typical MS brain, and cervical spinal cord lesions in MRI with contrast enhancement. Despite being positive for AQP4 he did not fulfill criteria for NMOSDs reflecting a borderline or atypical presentation of MS rather than NMO.

Current study had the limitation of small sample size. Nevertheless, considering the expected low prevalence of MS in the South Asia and very strict recruitment criteria of the present study, we have studied a more valid sample of MS as opposed to most of the other South Asian studies.

In conclusion, this is the first study done in Sri Lanka to meticulously characterize multiple sclerosis both clinically and paraclinically taking into consideration both OCBs and AQP4 antibody status. This group of Sri Lankan MS patients show similarities to typical Caucasian MS patients clinically and paraclinically. Nevertheless, their OCB positivity is lower compared to Caucasian MS populations.

## Figures and Tables

**Table 1:** Demographic characteristics.

Clinical characteristics	MS *n* (%)
Total	**69**
Male : female ratio	1 : 1.7
Mean age (range) years	34 (16–58)
Mean age at onset (range) years	32 (16–57)
Mean disease duration (range) years	3 (1–18)
Clinical subtypes (%)	
RRMS	37 (54)
SPMS	4 (6)
PPMS	1 (1)
CIS	27 (39)
Attack frequency (per year ± SD)	1.2 ± 0.5
Mean EDSS (range)	2.5 (0–8)
Upgraded Global MSSS	5.7 ± 2.6
Global ARMSSS	5.3 ± 2.9
History of travel to high MS prevalent country	2 (3)
Family history of MS	0

MS: multiple sclerosis; RRMS: relapsing remitting multiple sclerosis; SPMS: secondary progressive multiple sclerosis; PPMS: primary progressive multiple sclerosis; CIS: clinically isolated syndrome; SD: standard deviation; EDSS: Kurtzke expanded disability status scale; MSSS: multiple sclerosis severity score; ARMSSS: age related multiple sclerosis severity score.

**Table 2:** Clinical characteristics.

Clinical characteristics	MS *n* (%)
*Total*	*69 (100)*
*Optic neuritis*	
*Total*	*31 (45)*
Monocular	22 (32)
Binocular	9 (13)
Impaired visual acuity	31 (45)
Complete loss of light perception	0
Periorbital pain with eye movements	17 (25)
*Internuclear ophthalmoplegia*	17 (25)
*Cerebral sensory*	
*Total*	*31 (45)*
Regional distribution	
Right upper limb	19 (27)
Left upper limb	13 (19)
Right lower limb	18 (27)
Left lower limb	17 (24)
Trunk, right and left	7 (10)
Modalities	
Paraesthesia	22 (32)
Anaesthesia	27 (39)
Pain	18 (26)
*Cerebral motor*	
*Total*	*34 (50)*
Regional distribution	
Right upper limb	21 (31)
Left upper limb	11 (16)
Right lower limb	29 (42)
Left lower limb	23 (25)
*Cerebellar*	
*Total*	*22 (32)*
Ataxia	22 (32)
Dysarthria	5 (8)
Tremor	17 (24)
Past pointing	18 (26)
Vertigo	18 (26)
Nystagmus	12 (18)
*Brain stem/cranial nerves*	*20 (29)*
*Spinal cord*	
*Total*	*13 (19)*
Both sensory and motor	08 (12)
Sensory only	03 (4)
Motor only	02 (3)
*Autonomic*	
*Total*	*5 (8)*
Urinary incontinence	4 (6)
Faecal incontinence	3 (5)
*Transverse myelitis*	1 (1)
*Hearing loss *	3 (5)
*Lower motor facial nerve palsy*	5 (8)
*Fatigue *	33 (48)
*Ancillary symptoms *	27 (39)
Lhermitte's sign	10 (14)

All the symptoms were supported by clinical examination findings in all the attacks (up to a maximum of 5 attacks were included).

**Table 3:** Psychosocial features.

Psychosocial features (%)	Total MS (*n* = 69) *n* (%)
Psychological status	
Diagnosed depression	11 (16)
Low mood	38 (56)
Lack of self-esteem	25 (37)
Suicidal thoughts	11 (16)
Suicidal attempts	0
Sexual problems	
Impotence	5 (8)
Reduced libido	13 (19)
Divorced due to disease	2 (3)
Marital problems related to sexual activities	20 (30)
Studies affected	3 (5)
Lost job due to disability	16 (24)
Self-employed	3 (5)

**Table 4:** Magnetic resonance imaging characteristics.

*Number of MRIs analyzed (%)*	
Brain	143 (207)
Spinal cord (full)	27 (39)
Cervical cord only	03 (4)
*Types of MRI lesions (%)*	
Hyperintense lesions in T2W and FLAIR	69 (100)
Hypointense lesions in T1W	69 (100)
Number of brain lesions: average (range)	6 (3–12)
*Locations of lesions (%)*	
Periventricular	55 (80)
Juxtacortical	48 (69)
Infratentorial	51 (74)
Spinal cord^*∗*^(a)	11 (16)
Cervical cord (b)^*∗*^	12 (17)
Corpus callosum	34 (49)
Optic nerve	18 (26)

MRI: magnetic resonance imaging; T1W: T1 weighted pulse sequence; T2W: T2 weighted pulse sequence; (a) lesions of the spinal cord +/− involvement of cervical cord; (b) lesions confined to cervical cord. ^*∗*^Patients with T2W hyperintense spinal cord lesions which are centrally located and extend more than 3 spinal segments were excluded.

**Table 5:** Optic neuritis features and P100 response in VEP.

	*Number of eyes (%)*

*Clinical features of optic neuritis present*	31 (45)
Monocular	22 (32)
Binocular	9 (13)
*Availability of VEP reports*	40 (58)
*P100 response of VEP*	
Bilateral delay	33 (48)
Unilateral delay	6 (3)
Bilaterally normal	1 (1)
Right eye latency (ms)	110.60 (±34.6)
Right eye amplitude (*µ*V)	8.87 (±4.2)
Left eye latency (ms)	108.10 (±34.0)
Left eye amplitude (*µ*V)	8.76 (±3.98)

VEP: visual evoked potential.

**Table 6:** Cerebrospinal fluid.

CSF cell count (per mm3)^*∗*^	26 (±12)
Lymphocytes (%)^*∗*^	92 (±8.0)
Protein (mg/dl)^*∗*^	60.4 (±19.2)
Oligoclonal bands positivity	29 (42%)
AQP4 positivity	1 (1%)

CSF: cerebrospinal fluid; MS: multiple sclerosis; mm3: cubic millimetre; mg/dl: milligram per deciliter. ^*∗*^Values are presented as mean ± SD.

## Data Availability

The datasets used and/or analyzed during the current study are available from the corresponding author on reasonable request.

## Abbreviations

MS: Multiple sclerosis
MRI: Magnetic resonance imaging
OCB: Oligoclonal bands
NMOSD: Neuromyelitis spectrum disorder
VEP: Visual evoked potential
T1W: T1 weighted
T2W: T2 weighted
FLAIR: Fluid-attenuated inversion recovery
CSF: Cerebrospinal fluid
AQP4: Aquaporin-4
ADEM: Acute disseminated encephalomyelitis
IEF and IB: Iso electric focusing and immunoblotting
RRMS: Relapsing remitting multiple sclerosis
SPMS: Secondary progressive multiple sclerosis
PPMS: Primary progressive multiple sclerosis
CIS: Clinically isolated syndrome
CNS: Central nervous system
EDSS: Expanded disability status scale
NMO: Neuromyelitis optica
ON: Optic neuritis.
